# The application of DM-MSTP method on Tunisian financial market: 2024 case study

**DOI:** 10.3389/frai.2026.1776919

**Published:** 2026-04-29

**Authors:** Souhail Dhouib, Mouna Abdelhedi, Siwar Ellouz, Hanene Ezzine, Habib Chabchoub

**Affiliations:** 1 Higher Institute of Industrial Management of Sfax, University of Sfax, Sfax, Tunisia; 2 Finance Department, Higher School of Business of Sfax, University of Sfax, Sfax, Tunisia; 3 College of Business Al Ain, Al Ain University, Abu Dhabi, United Arab Emirates

**Keywords:** artificial intelligence, financial clustering, Minimum Spanning Tree Problem, operations research, optimization

## Abstract

The Novel Dhouib Matrix Minimum Spanning Tree Problem (DM-MSTP) method is used to illustrate a topological relationship between all Tunisian financial market for 2024. The results reveal a clear hierarchical organization with the financial sector playing a central bridging role, particularly through banks and financial services, which connect consumer-oriented and real-economy sectors. A two-cluster configuration highlights a tightly integrated group of cyclical sectors basic materials, construction and building materials, and industry characterized by strong co-movements and limited diversification potential. Extending the analysis to a three-cluster framework provides finer granularity, separating consumer and service sectors, real-economy production sectors, and financial intermediaries into distinct groups. These partitioning underscores differential channels of shock transmission, with faster contagion within cyclical sectors and more buffered dynamics among diversified service sectors.

## 1 Introduction

In recent decades, financial markets have undergone profound transformations driven by globalization, technological progress, and continuous financial innovation. These developments have increased market dynamism and interconnectedness, transforming financial systems into complex networks of institutions, assets, and sectors. As a result, shocks originating in one segment of the market can propagate rapidly across the entire system, amplifying systemic risk. Understanding these interdependencies has therefore become essential for ensuring financial stability, improving risk management, enhancing portfolio diversification, and designing effective regulatory frameworks (Mantegna, 1999; Tumminello et al., 2005).

Traditional financial analysis methods, including correlation analysis, regression models, and factor-based approaches, provide useful insights into linear relationships among assets. However, these techniques often fail to capture the nonlinear, high-dimensional, and hierarchical structures that characterize modern financial markets (Kenett et al., 2010). Financial systems are influenced by multiple interacting forces such as investor behavior, institutional constraints, sectoral dynamics, and macroeconomic shocks leading to complex dependency patterns that are not easily detected using conventional econometric tools. Moreover, these approaches typically overlook indirect relationships and network-wide propagation mechanisms, limiting their effectiveness in analyzing systemic risk.

In this context, complex network theory has emerged as a powerful framework for modeling and analyzing financial markets. By representing markets as graphs in which nodes correspond to assets or institutions and edges capture their interdependencies, network-based approaches allow for the identification of both direct and indirect relationships. These methods enable researchers to detect clusters of interconnected assets, uncover hierarchical structures, and identify systemically important nodes within the market (Bonanno et al., 2004; Onnela et al., 2003). As such, network analysis provides a more comprehensive understanding of financial market structure compared to traditional techniques.

Among network-based methodologies, the Minimum Spanning Tree (MST) approach has gained particular prominence. By selecting the *N* − 1 most significant connections among N assets, the MST filters out noise and reveals the backbone of market connectivity (Mantegna, 1999). This approach has been widely applied across various financial markets, including equities, foreign exchange, and fixed income, offering valuable insights into sectoral clustering, market organization, and risk transmission. Recent studies continue to confirm its relevance, demonstrating its ability to uncover meaningful structural patterns and diversification opportunities in both developed and emerging markets.

Despite its usefulness, conventional MST methodologies present several limitations. Their construction often depends on parameter choices such as distance metrics or filtering thresholds—which may introduce subjectivity and affect the stability of the resulting network. In addition, MST structures can be sensitive to noise and estimation errors in correlation matrices, potentially reducing the reliability of inferred relationships. These challenges are particularly pronounced in high-dimensional or rapidly evolving financial environments.

To address these limitations, Dhouib et al. (2024) introduced the Dhouib-Matrix Minimum Spanning Tree Problem (DM-MSTP), a metaheuristic framework designed to construct financial networks in a more robust and efficient manner. The DM-MSTP approach relies on correlation-based distances to extract the essential structure of financial markets while minimizing the impact of noise and eliminating the need for multiple tuning parameters. By focusing on the most informative connections, it provides a stable and interpretable representation of market topology, making it particularly suitable for analyzing complex financial systems.

Emerging markets provide a particularly relevant setting for applying network-based methodologies, as their structural characteristics differ significantly from those of developed economies. These markets are typically characterized by lower liquidity, higher concentration, and evolving regulatory environments, making them more susceptible to volatility and contagion effects. The Tunisian stock market represents a compelling case in this regard, combining moderate liquidity, a limited number of listed firms, and gradual integration into global financial systems. Despite its importance, empirical research examining the Tunisian market from a network perspective remains limited, especially when using advanced computational approaches.

This study aims to bridge this gap by applying the DM-MSTP framework to analyze the structural topology of the Tunisian stock market. Specifically, it seeks to (i) identify the key nodes that play a central role in market dynamics, (ii) detect sectoral clustering patterns that inform diversification strategies, and (iii) provide a clearer representation of financial interdependencies by filtering out noisy or weak connections.

Accordingly, this paper contributes to the literature in three main ways. First, it applies the recently developed DM-MSTP methodology to an emerging market context, providing new empirical evidence on financial network structure in Tunisia. Second, it offers insights into sectoral clustering, centrality, and hierarchical organization within the Tunisian market, highlighting the role of key sectors in shaping market dynamics. Third, it demonstrates the advantages of parameter-free metaheuristic approaches in improving the robustness and interpretability of financial network models.

The remainder of the paper is organized as follows. Section 2 reviews the relevant literature on financial networks and MST-based methodologies. Section 3 presents the DM-MSTP framework and its theoretical foundations. Section 4 describes the data and empirical methodology. Section 5 discusses the empirical results, and Section 6 concludes with implications for investors, regulators, and future research.

## 2 Literature review

The rapid expansion of research on financial networks has reshaped how scholars understand market structure, systemic fragility, and the transmission of shocks across interconnected assets. In contrast with traditional econometric approaches that rely primarily on pairwise correlations or linear regressions, network-based methods provide a systemic representation of financial markets by modeling assets as nodes and their statistical or causal relationships as edges. This perspective allows for the detection of hidden structures, hierarchical dependencies, and channels of contagion that remain invisible in classical models (Newman, 2003). The literature has established that financial markets resemble complex adaptive systems, where interactions between components often display nonlinearity, heterogeneity, and time-varying intensity, all features that justify the adoption of network-theoretic tools.

A significant body of work has investigated the statistical properties of correlation matrices used to derive financial networks. Early contributions demonstrated that empirical correlation matrices of asset returns are heavily contaminated by noise, especially in markets with many assets and limited time-series observations. Random Matrix Theory (RMT) emerged as a foundational framework to filter out spurious eigenmodes, enabling more reliable identification of true market structure (Laloux et al., 1999; Plerou et al., 1999). Later studies showed that such filtering strengthens the robustness of the topological features extracted from networks and improves the stability of Minimum Spanning Trees (MSTs) derived from correlation distances (Bouchaud and Potters, 2000).

The MST method has become one of the most widely implemented tools for simplifying dense financial networks into interpretable topological skeletons. By retaining only, the *N* − 1 strongest connections among *N* assets, MSTs highlight the hierarchical backbone of market dependencies, revealing clusters that often correspond to economic sectors or institutional groups (Jang and Lee, 2011; Dhouib et al., 2024). Applications demonstrate that MSTs are sensitive to major market events, with topological structures reorganizing during crises and periods of instability (Preis et al., 2013).

In recent years, the analysis of financial markets has increasingly relied on network filtering techniques to extract meaningful structures from dense correlation matrices. Among these approaches, the Minimum Spanning Tree (MST) has become one of the most widely used methods due to its simplicity and interpretability. By retaining only *N* − 1 links, MST extracts the backbone of the financial network and highlights the most significant relationships between assets or sectors (Mantegna, 1999). However, this strong reduction of information also implies that potentially relevant connections are discarded, which may limit the ability of MST to capture the full complexity of financial interdependencies.

To address this limitation, several alternative filtering methods have been proposed in the literature. The Planar Maximally Filtered Graph (PMFG) extends the MST by allowing a higher number of edges while preserving planarity constraints, thereby enabling the representation of more complex structures such as loops and higher-order interactions. Similarly, the Triangulated Maximally Filtered Graph (TMFG) further generalizes this idea by constructing a triangulated network that maintains a balance between information retention and structural sparsity. These approaches provide a richer representation of financial systems compared to MST, as they preserve additional economically meaningful connections that are otherwise eliminated in tree-based structures.

Another class of methods relies on threshold-based networks, where edges are retained if their correlation exceeds a predefined threshold. This approach offers flexibility in controlling network density; however, it is highly sensitive to the choice of the threshold parameter, which may lead to instability in the resulting network structure and reduce comparability across studies.

In this context, MST-based approaches remain particularly attractive for their robustness, simplicity, and ability to filter noise in high-dimensional financial data. The DM-MSTP method adopted in this study belongs to this family of MST-based techniques. It preserves the essential *N* − 1 structure of the network while improving the efficiency and stability of the construction process. Compared to classical MST algorithms, DM-MSTP provides a structured matrix-based formulation that enhances computational handling and facilitates clearer extraction of the market backbone without the need for parameter tuning.

Recent comparative studies, such as Esmalifalak and Moradi-Motlagh (2025), provide a systematic evaluation of MST, PMFG, TMFG, and related filtering approaches, highlighting their respective strengths and limitations in capturing financial network topology. These contributions suggest that no single method is universally optimal; rather, the choice of filtering technique depends on the trade-off between interpretability, complexity, and information retention. In line with this perspective, the present study positions DM-MSTP as a parsimonious and robust MST-based framework particularly suitable for emerging markets, where data noise and structural instability are more pronounced.

Despite their interpretability, MSTs may omit economically relevant secondary links, motivating complementary techniques such as bootstrap-based edge reliability tests and the extraction of statistically validated backbones (Serrano et al., 2009). Recent work further enhances MST stability by adjusting for time-scale effects and filtering out short-horizon noise, a recurring issue in high-frequency and daily financial datasets (Kwapień et al., 2017). Li et al. (2024) develop a network-augmented portfolio selection model to distinguish between long- and short-run correlation structures in financial markets. Their findings show a positive influence of the long-run correlation network and the negative influence of the short-run correlation network on portfolio weights. Choi and Kim (2025) employ multiple dependence measures and network filtering techniques (MST, PMFG, ALMST, and *p*-value-based networks) to uncover discrepancies in sector-based financial networks. Their findings demonstrate that relying on a single metric may mask important dimensions of market interdependencies, highlight.

ting the need for a multifaceted analytical framework. Engsig et al. (2024) introduce DomiRank centrality that integrates local and global topological information to identify dominant nodes and structurally fragile regions within complex networks. The study shows that DomiRank outperforms traditional centrality measures in detecting critical nodes and assessing network vulnerability and resilience. Esmalifalak and Moradi-Motlagh (2025) provides a comprehensive review of correlation network methodologies in economics and finance, highlighting the central role of filtering techniques such as the Minimum Spanning Tree (MST), Planar Maximally Filtered Graph (PMFG), and related approaches in extracting meaningful financial interdependencies. It further underlines the increasing interconnectedness of global financial markets and the importance of time-varying dynamics in understanding systemic risk and market structure. This author indicates that integration of advanced tools is required in network analysis.

Beyond simple correlation networks, scholars have emphasized the need to distinguish genuine structural dependencies from indirect or spurious connections. Partial-correlation networks, sparse graphical models, and factor-adjusted econometric networks allow researchers to recover direct relationships and identify core-periphery structures more accurately than raw correlation-based methods (Barigozzi and Brownlees, 2019). In parallel, the systemic risk literature has shifted toward modeling directional and dynamic connectedness. Measures based on variance decompositions (Diebold and Yilmaz, 2014) and interconnectedness metrics for the finance, insurance sector (Billio et al., 2012) provide insights into shock propagation mechanisms, complementing the static representation of MST-based networks. Lee et al. (2025) apply symmetry-aware graph neural networks (GCN and GAT) to model financial market dependencies and improve return prediction across global indices during crises period. Their results show that incorporating symmetric and bidirectional spillover structures enhances predictive performance, particularly during periods of market instability and systemic risk. Samal et al. (2021) integrate geometry-based network measures, such as Ricci curvature, to capture higher-order interactions and structural properties of financial systems beyond pairwise correlations. Their results show that these geometric indicators effectively detect periods of market instability and distinguish between normal and crisis regimes.

A related but distinct research stream examines contagion and stability using exposure-based or interbank networks. These models highlight how network topology, such as connectivity, assortative, and degree concentration, fundamentally shapes the propagation of failures and liquidity shocks. Seminal contributions such as the clearing model of Eisenberg and Noe (2001), the contagion simulations of Gai and Kapadia (2010), and the systemic-risk analysis of Amini et al. (2012) illustrate how even small shocks can amplify when networks contain structural vulnerabilities. A broader systems perspective links financial fragility to patterns observed in ecological networks (Haldane and May, 2011), reinforcing the argument that network sparsity, modularity, and centrality critically determine resilience.

In foreign exchange and stock markets alike, network applications extend to investigating cross-market integration, contagion channels, and the role of dominant hubs. Studies on currency networks (Mizuno et al., 2006) and international equity networks (Preis et al., 2013) demonstrate the value of MSTs for analyzing global interdependence. Emerging and frontier markets exhibit distinctive network topologies, often shaped by liquidity constraints, sectoral concentration, and institutional characteristics. These structural features justify more advanced MST-based techniques, especially methods that improve parameter robustness and computational efficiency, for markets such as Tunisia, where traditional models may fail to capture subtle yet economically significant interconnections.

Recent advances have integrated machine learning and optimization into network construction. Metaheuristics, sparse estimation techniques, and data-driven filtering approaches offer promising improvements in handling nonlinearity and high dimensionality. Such developments, Dhouib et al. (2024) introduces a novel algorithm the DM-MSTP, designed to overcome the limitations of conventional MST computation by enhancing noise reduction, parameter efficiency, and topological interpretability. Given the dynamic and often fragile nature of emerging markets, applying such an approach to the Tunisian financial system is both relevant and timely, providing a high-resolution mapping of market structure that can inform risk management, regulatory oversight, and diversification strategies.

## 3 The DM-MSTP method

Recent advances have integrated optimization-based procedures into network construction, particularly in the context of Minimum Spanning Tree (MST) extraction. In this line of research, DM-MSTP (Dhouib, 2024) is proposed as an MST-based computational procedure aimed at improving the efficiency and stability of network construction in large financial datasets. Rather than introducing a fundamentally new methodological framework, the approach should be understood as a refined implementation of classical MST algorithms, grounded in standard graph optimization techniques such as those of Prim and Kruskal.

The main contribution of DM-MSTP lies in its algorithmic refinement of the MST construction process, particularly by enhancing computational efficiency, reducing numerical instability, and improving the handling of noisy high-dimensional correlation matrices. It therefore does not alter the theoretical foundations of MST, but rather optimizes its practical execution to ensure more stable and reproducible network representations.

Given the complexity and noise typically characterizing financial data, especially in emerging markets such as Tunisia, this refined MST-based implementation may offer practical advantages in terms of robustness and interpretability. Accordingly, DM-MSTP is used in this study as an operational enhancement of the classical MST framework, rather than as a novel methodological contribution.

At its core, the DM-MSTP method represents financial indices as a weighted graph, where each index corresponds to a node and edges reflect relationships derived from the correlation matrix. Correlations are transformed into distances using a standard transformation, such that stronger correlations correspond to shorter distances and weaker correlations correspond to longer distances.

**Figure 1:**
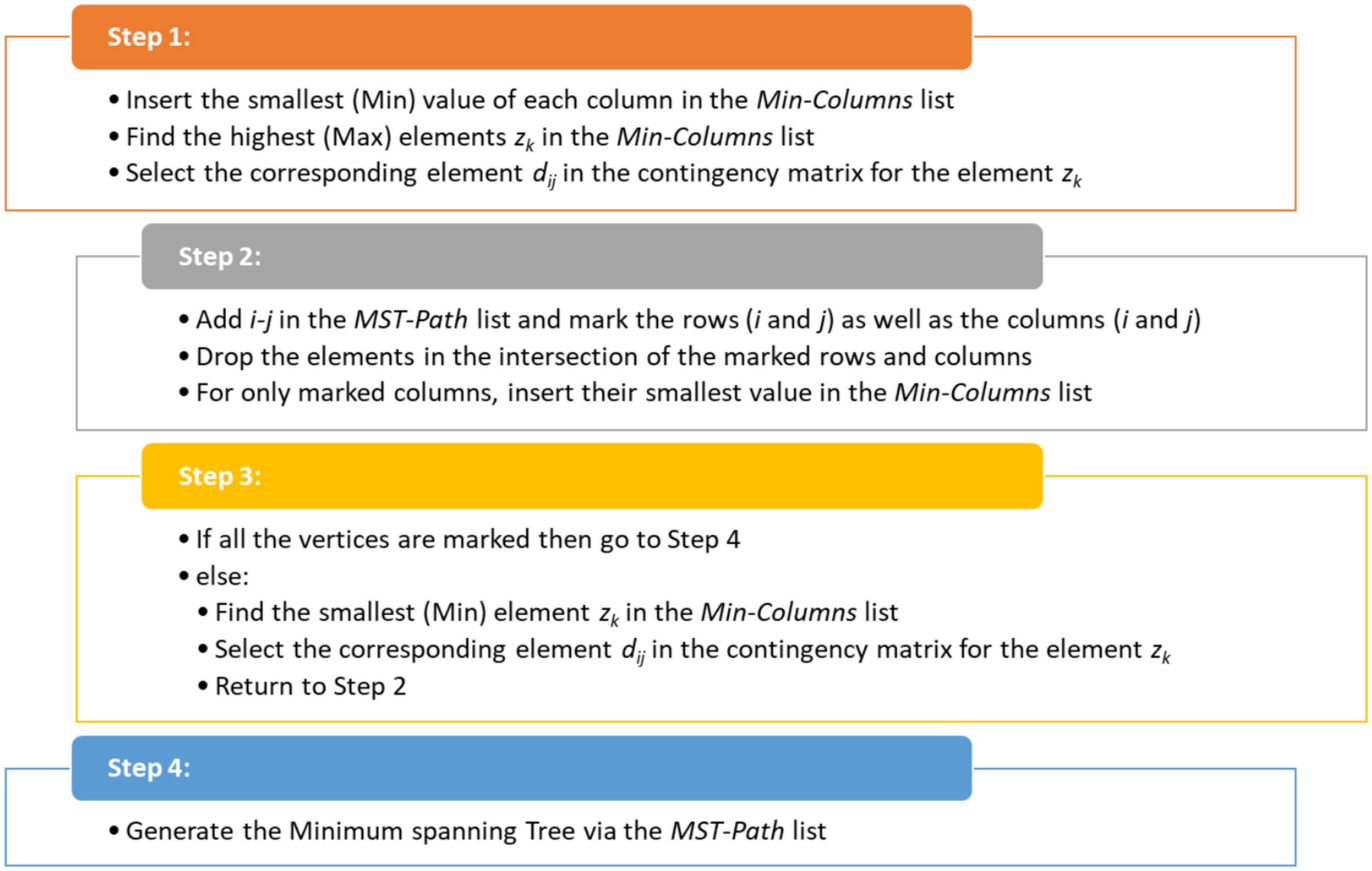
The general structure of DM-MSTP.

In addition, DM-MSTP follows a structured and parameter-free procedure that focuses on extracting the *N* − 1 most relevant connections, thereby reducing noise and improving interpretability compared to full correlation matrices. The procedure consists of four main steps, as illustrated in Figure 1.

DM-MSTP belong to the novel concept of Artificial Intelligence entitled Dhouib-Matrix (DM) where several other methods are invented such as the DM-TSP1 to solve the Traveling Salesmen Problem (Miledi et al., 2025; Dhouib et al., 2025a; Dhouib et al., 2025b), the DM-TP1 to unravel the Transportation Problem (Dhouib et al., 2025b, c), the DM-SPP to plan the shortest path between two mobile robot positions (Dhouib, 2025a; Dhouib et al., 2026; Dhouib and Chabchoub, 2026) and the DM4 metaheuristic in (Dhouib, 2025b; Dhouib et al., 2025d).

## 4 Step by step application of DM-MSTP on Tunisian financial market

In this section the 2024 Tunisian financial market data is analyzed using a novel clustering technique namely DM-MSTP. The proposed method DM-MSTP is an unsupervised technique requiring zero parameters.

**Figure 2:**
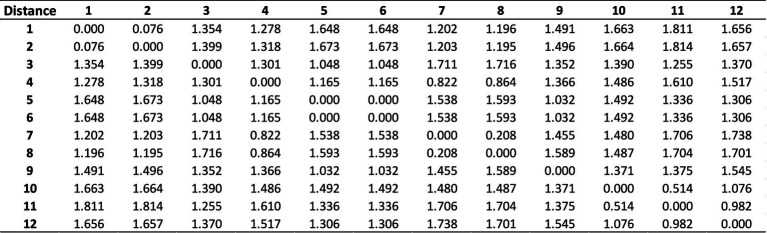
Modified correlation matrix for Tunisian financial stocks.

Originally DM-MSTP finds the minimum spanning tree for an undirected weighted graph. To adapt DM-MSTP to financial market, each index will be represented as a node and the covariance matrix between all indices represent the vertices weights. In this paper, the Tunisian financial stocks for 2024 will be considered where 12 sectorial indices are represented (see Figure 2): The Financial Companies is coded by 1, the Banks by 2, the Insurance by 3, the Financial Services by 4, the Consumers Services by 5, the Distribution by 6, the Consumer Goods by 7, the Food and Bevery by 8, the Industry Household Products and Personal Care by 9, the Industry by 10, the Construction and Building Material by 11 and the Basic Materials by 12.

This sector-based approach allows for a clearer identification of the underlying economic structure of the market (Choi and Kim, 2025) by emphasizing inter-sectoral linkages. By focusing on aggregated sectoral indices, the analysis captures the dominant transmission channels across key economic segments, which is particularly relevant in emerging markets where macroeconomic and sectoral dynamics often prevail over idiosyncratic firm-level effects.

To cluster the input data DM-MSTP starts by converting the covariance matrix (see Figure 2).

**Figure 3:**
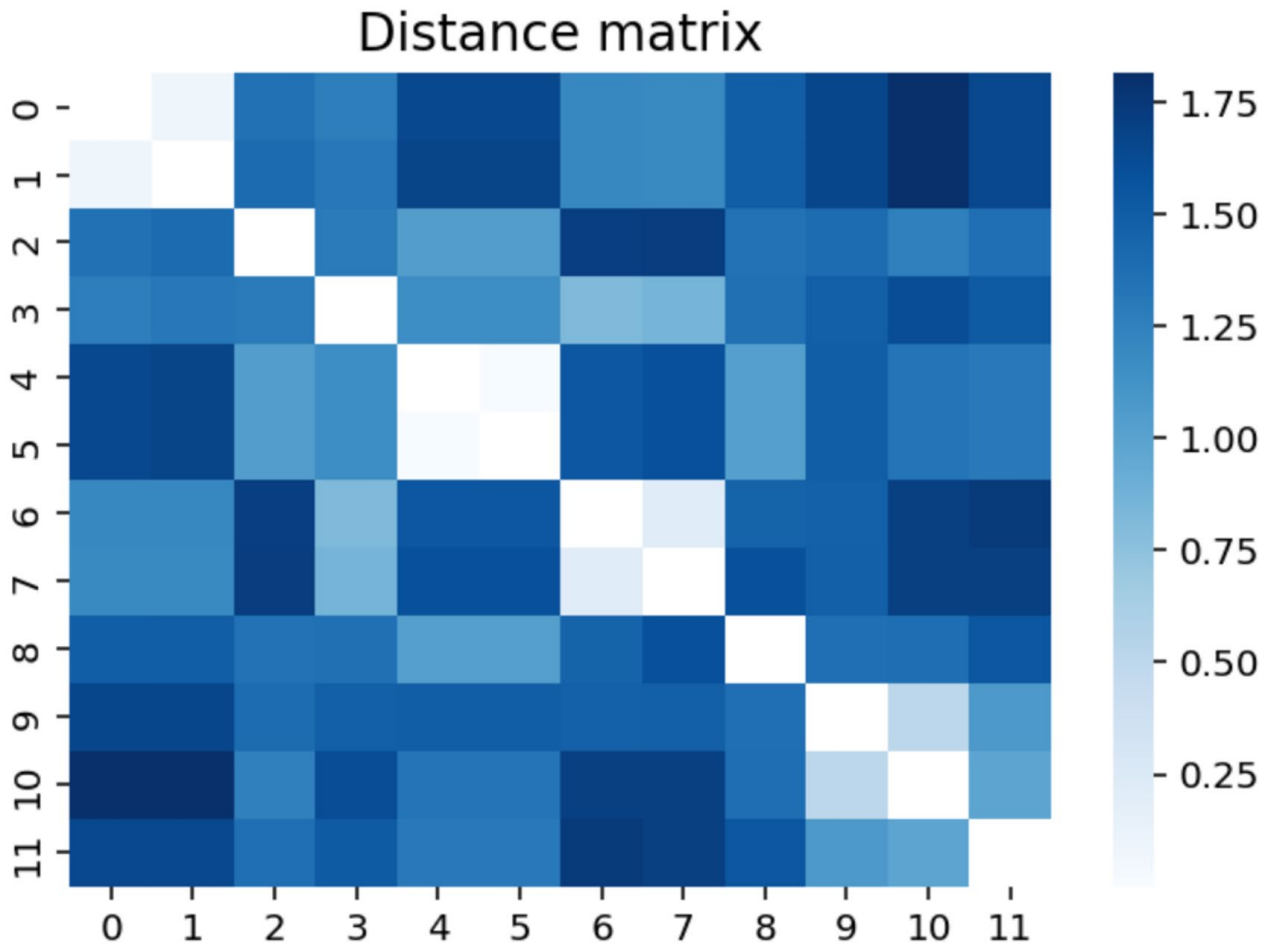
Python graphical representation of the modified correlation matrix.

For more clarification, the modified distance matrix is represented using Python programming language (see Figure 3).

**Figure 4:**
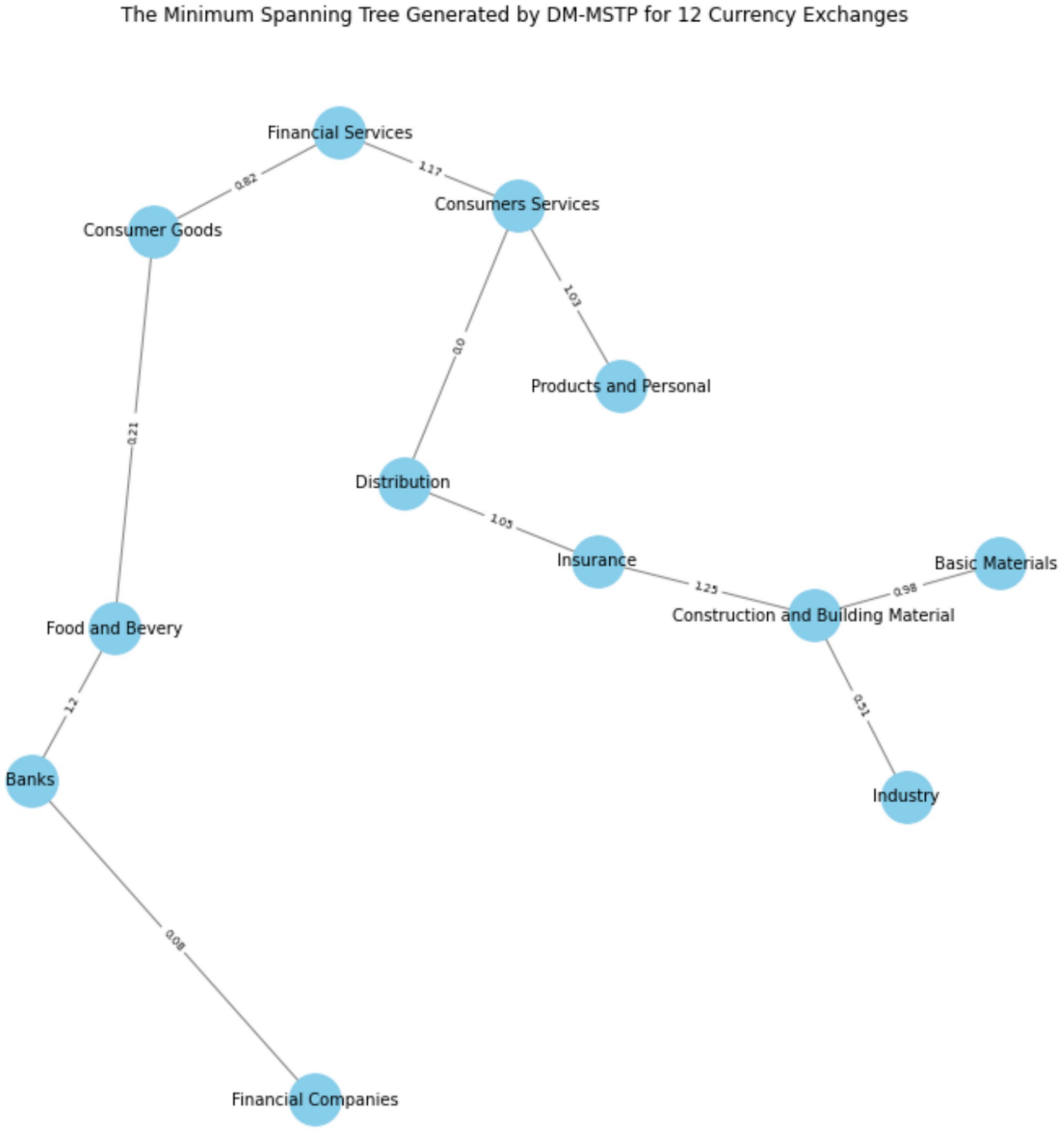
The minimum spanning tree generated by DM-MSTP.

Figure 4 illustrates the generated minimum spanning tree created by the DM-MSTP method where all nodes are connected. The corresponding distance between any two nodes is represented in the center of the edge.

Furthermore, to assist decision-makers in clustering the Tunisian financial market in 2024 into two distinct groups, the minimum spanning tree (MST) generated by DM-MSTP (presented in Figure 4) can be examined by removing the highest-distance link (1.25) connecting (i) the Insurance index, (ii) the Construction, and (iii) the Building Materials index. Figure 4 shows that the financial sector plays a central role in linking other sectors within the market. In particular, banks and financial services connect the food and beverage sector, while financial services also serve as a bridge between consumer services and consumer goods. In addition, the insurance sector links the distribution sector with construction and building materials. These results can be explained by the strategic role of banks and financial services firms in financing Tunisian companies, which remain highly dependent on bank-based funding, as well as by the dominant role of banks in supporting the national economy. Indeed, the banking sector has been among the most profitable sectors in the Tunisian financial market, particularly during the COVID-19 period. These findings are consistent with Ben Yaala and Henchiri (2025), who report that, in the post-COVID period, financial services, industrials, and consumer goods exhibit strong interconnections, reflecting the persistence of structural linkages. Moreover, using five Tunisian sectoral stock indices, namely Banking, Financial Services, Automobile, Industry, and Materials, Neifar (2020) documents significant volatility spillovers from the Materials sector to the Industrial sector, further supporting the existence of strong inter-sectoral dependence in the Tunisian stock market.

**Figure 5:**
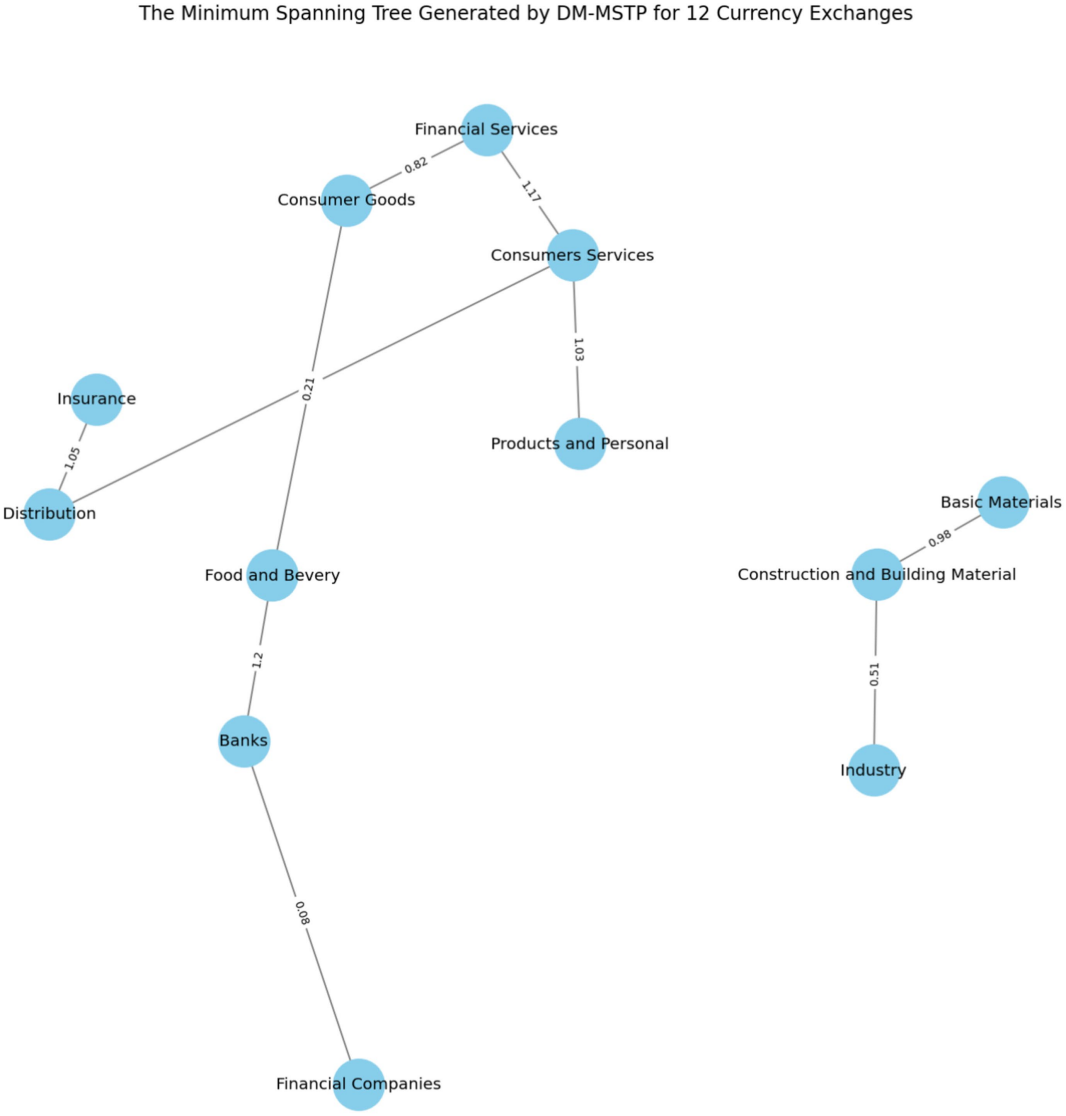
Clustering the Tunisian financial market into two groups.

Figure 5 represents the clustering of Tunisian financial market in two groups where the first group gather three indices (“Basic Material,” “Industry” and “Construction and Building Material”) and the second group fold the remainder nine indices.

Figure 5 illustrates the Minimum Spanning Tree (MST) topology of the Tunisian equity market, derived using the Dynamic Minimum Spanning Tree Protocol (DM-MSTP) applied to 12 sectoral indices. This network-based approach captures the most significant inter-sectoral linkages while minimizing redundancy, thereby revealing the hierarchical structure of co-movement and systemic interdependence.

The MST highlights a pronounced dichotomy in market organization. Cluster I comprises three closely connected cyclical sectors—Basic Materials, Construction and Building Materials, and Industry, linked by relatively short distances (0.98, 0.51), indicating strong fundamental or macroeconomic co-dependence. Such interconnections may reflect domestic demand cycles, commodity exposure, or infrastructure policies. These sectors form a tightly integrated subnetwork, suggesting limited diversification potential within the cluster.

Cluster II includes the remaining nine sectors, such as Financial Services, Consumer Goods, Banks, and Food and Beverage, forming a more loosely connected and structurally heterogeneous group. While exhibiting greater internal diversity, these sectors collectively respond to broader macro-financial factors (e.g., interest rates, inflation, global risk sentiment) rather than sector-specific shocks.

From a systemic risk perspective, this bifurcation implies differential propagation of volatility or contagion across clusters. Transmission is expected to be faster and more pronounced within Cluster I, whereas Cluster II, due to its internal heterogeneity, may act as a buffer or absorber.

These results are consistent with prior MST applications in emerging markets (e.g., Mantegna, 1999; Tumminello et al., 2010; Onnela et al., 2003) and provide novel insights into the Tunisian market’s structure, a small, illiquid, and policy-sensitive market where sectoral clustering can inform asset allocation, systemic risk assessment, and macroprudential policy design.

**Figure 6:**
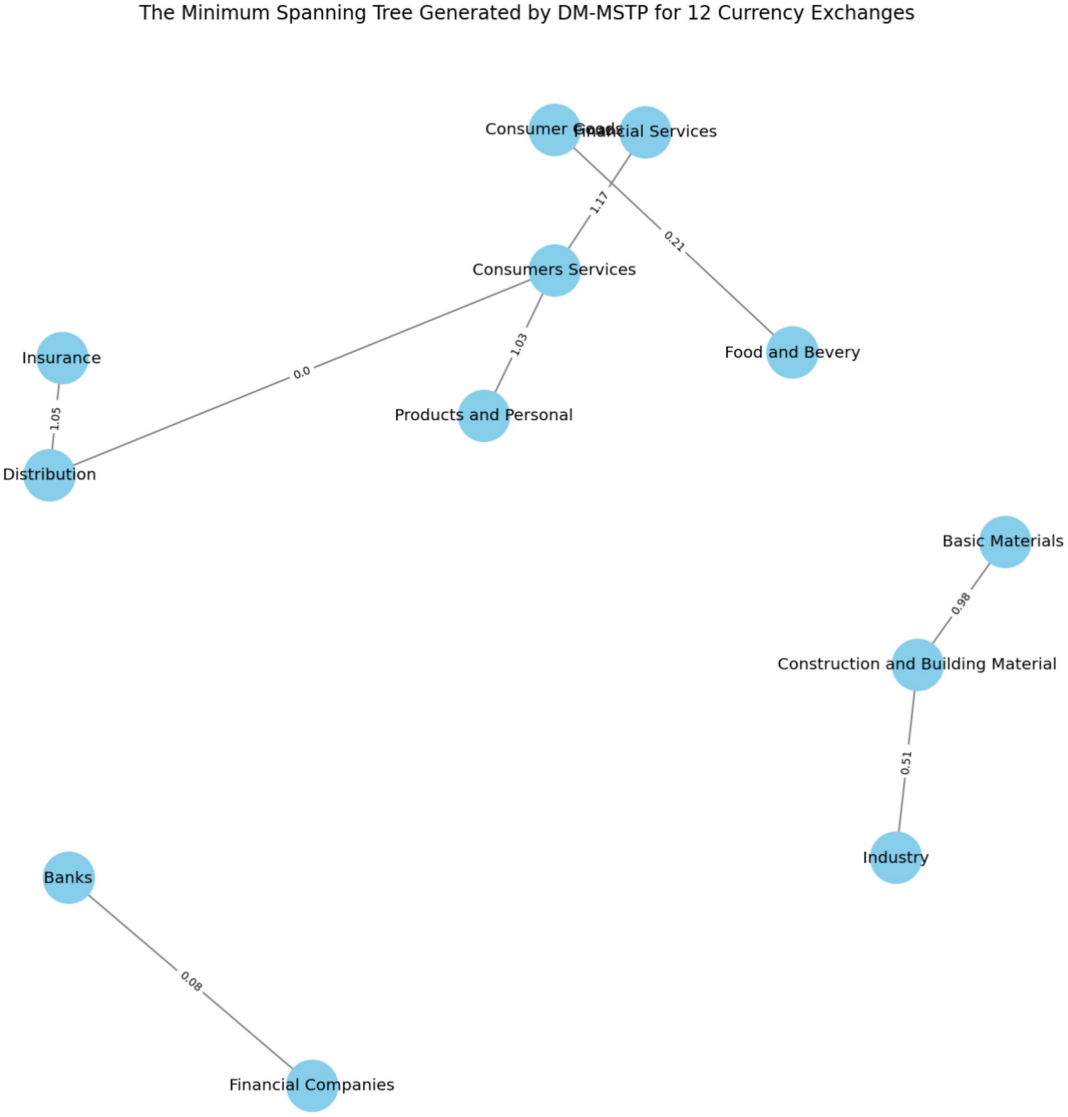
Clustering the Tunisian financial market into three groups.

The MST also supports flexible partitioning. For instance, applying a higher distance threshold (1.2) produces a three-cluster solution, offering finer granularity for portfolio construction or regulatory monitoring. This refinement can isolate financial intermediaries (e.g., Banks, Financial Companies) as distinct risk groups (see Figure 6).

Figure 6 shows that the Tunisian financial market divided into three main groups of sectors. The first group brings together consumer-oriented and service-related sectors, including consumer goods, consumer services, food and beverage, and financial services. The close connections among these sectors suggest that they tend to move together, likely reflecting the importance of domestic demand and service activities in shaping market dynamics.

The second group consists of production and real-economy sectors, such as basic materials, construction and building materials, and industry. The strong links within this group indicate that these sectors are influenced by common economic factors, particularly investment activity and infrastructure development, which drive their co-movements in the market.

The third group is mainly formed by financial institutions, represented by banks and financial companies, which appear more weakly connected to the other sectors. This relative separation suggests that the financial sector follows partly distinct dynamics, driven by regulatory conditions, profitability patterns, and financial intermediation activities.

Overall, this three-cluster structure highlights the diverse nature of the Tunisian financial market, where consumer demand, real-economy activity, and financial intermediation evolve with different but interconnected dynamics, offering useful insights for investors and policymakers.

## 5 Discussion of the empirical findings and insights

The application of the Dhouib Matrix Minimum Spanning Tree Problem (DM-MSTP) to the Tunisian financial market reveals a hierarchical and highly interconnected structure, capturing both sectoral dependencies and systemic vulnerabilities. Banks and financial services emerge as central hubs, bridging otherwise fragmented segments of the market. Their dominant position reflects the bank-based nature of the Tunisian economy, where firms rely heavily on bank financing for both operational and investment activities. As a result, disturbances affecting the banking sector can rapidly propagate across the entire market, amplifying systemic risk.

A tightly interconnected cluster composed of Basic Materials, Industry, and Construction highlights strong production linkages within the real economy. The short topological distances between these sectors indicate that shocks such as fluctuations in commodity prices, supply chain disruptions, or changes in infrastructure spending are transmitted rapidly, generating significant volatility spillovers. This high degree of interconnectedness reduces the system’s capacity to absorb shocks, making this cluster particularly vulnerable to both domestic demand fluctuations and external economic pressures.

In contrast, a more heterogeneous cluster including Financial Services, Consumer Goods, and Banks exhibits looser interconnections and greater sensitivity to macro-financial variables such as interest rates, inflation, and liquidity conditions. This structural diversity enhances the cluster’s ability to absorb shocks, thereby contributing to overall market stability. The coexistence of tightly connected production clusters and more diversified, loosely connected groups illustrates how network heterogeneity can mitigate the transmission of systemic risk.

From a methodological perspective, the DM-MSTP approach provides a clear and robust representation of financial interdependencies by isolating the most significant connections within the network. This feature is particularly valuable in emerging markets, where low liquidity and irregular trading activity may distort conventional correlation-based measures. By emphasizing the essential structure of the market, the method enables the identification of key nodes and critical clusters, offering valuable insights into potential contagion channels.

The findings demonstrate that financial stability in Tunisia depends not only on the performance of individual sectors but also on the structure of interconnections across the market. The network topology highlights both zones of vulnerability where shocks propagate rapidly and stabilizing segments that help absorb disturbances. These insights provide a useful foundation for risk monitoring, portfolio diversification, and the design of targeted macroprudential policies aimed at strengthening market resilience.

## 6 Conclusion

The 2024 case study of the Tunisian financial market illustrates the effectiveness of the Dhouib Matrix Minimum Spanning Tree Problem (DM-MSTP) in uncovering the intricate structures of emerging economies. By modeling the market as a network of interconnected sectoral nodes, the DM-MSTP produces a detailed, hierarchical representation of financial interdependencies, going beyond the limits of traditional linear econometric methods. This approach reveals the market’s internal organization, sectoral clustering, and systemic risk, providing valuable insights for investors, regulators, and policymakers.

The empirical analysis highlights a distinct bifurcation in the structure of the Tunisian market. At its core, the Cyclical Core comprising sectors such as Basic Materials, Construction and Building Materials, and Industry exhibits tight interconnections, reflecting a high degree of co-dependence driven by common macroeconomic forces, including infrastructure policies and domestic demand cycles. This core represents a zone of elevated contagion risk, as shocks to any one sector are likely to propagate rapidly across the others, limiting opportunities for internal diversification. In contrast, the Heterogeneous Majority, which includes sectors such as Financial Services, Banks, and Consumer Goods, is more loosely connected and structurally diverse. While these sectors remain influenced by broad macro-financial trends, their relative heterogeneity allows them to function as a stabilizing buffer against sector-specific shocks.

The DM-MSTP also offers the flexibility to conduct more granular analyses. By adjusting the distance threshold, Banks and Financial Companies can be separated into a distinct third cluster, providing regulators with a focused view of systemic risk within the financial intermediary sector. This level of insight is particularly valuable for macroprudential supervision and for monitoring potential channels of contagion. For investors and portfolio managers, the findings serve as a practical “diversification map,” enabling asset selection across different clusters to reduce portfolio risk effectively. Avoiding over-concentration in the Cyclical Core minimizes exposure to localized shocks and enhances overall portfolio resilience. For policymakers and regulators, the network structure offers a diagnostic perspective on systemic vulnerabilities, highlighting sectors where interventions could produce amplified effects across the broader economy. The tightly linked industrial and construction sectors illustrate the potential impact of targeted policy measures, while the heterogeneous majority functions as a stabilizing force during periods of market volatility.

From a broader perspective, the analysis emphasizes the interconnectedness of emerging financial markets. Understanding sectoral dependencies and the market’s network backbone can guide investment strategies and policy decisions, helping stakeholders anticipate and mitigate the effects of systemic shocks. By mapping these interconnections, the DM-MSTP provides a practical framework for monitoring financial stability and identifying potential points of vulnerability, which is particularly critical in markets characterized by moderate liquidity and concentrated sectoral participation.

Our research also highlights important considerations regarding the evolving dynamics of the Tunisian market. As global economic conditions fluctuate, political transitions occur, or domestic policies are implemented, the configuration of sectoral linkages is likely to shift. Tracking these changes over time allows investors, regulators, and policymakers to adjust strategies proactively, thereby enhancing market resilience. Moreover, integrating broader financial indices and cross-border connections could shed light on Tunisia’s increasing integration into global financial networks, a factor that is crucial for long-term economic planning and investment decision-making.

The application of the DM-MSTP to the 2024 Tunisian financial market provides a clear understanding of sectoral interdependencies, systemic risk patterns, and the hierarchical organization of the market. For investors, it supports more informed portfolio decisions and effective risk management, for regulators, it identifies critical vulnerabilities and guides macroprudential oversight; and for policymakers, it clarifies the potential ripple effects of targeted interventions. By uncovering the structural dynamics of the market, this approach underscores the importance of understanding interconnectedness in emerging economies and offers a practical framework to support financial stability, resilience, and informed decision-making as markets continue to grow in complexity and interconnectivity.

## Data Availability

Publicly available datasets were analyzed in this study. This data can be found at: https://www.bvmt.com.tn/fr/rapports-activites.
